# Pre-pregnancy blood pressure and pregnancy outcomes: a nationwide population-based study

**DOI:** 10.1186/s12884-022-04573-7

**Published:** 2022-03-19

**Authors:** Young Mi Jung, Gyu Chul Oh, Eunjin Noh, Hae-Young Lee, Min-Jeong Oh, Joong Shin Park, Jong Kwan Jun, Seung Mi Lee, Geum Joon Cho

**Affiliations:** 1 Department of Obstetrics and Gynecology, Seoul National University College of Medicine, 101 Daehak-ro, Jongno-gu, Seoul, 03080 South Korea; 2 Department of Internal Medicine, Seoul National University College of Medicine, Seoul, South Korea; 3 Department of Cardiology, Seoul St. Mary’s Hospital, The Catholic University of Korea, Seoul, South Korea; 4 Korean University Guro Hospital Samrt Healthcare Center, Seongbuk-gu, Seoul, South Korea; 5 Department of Obstetrics and Gynecology, Guro Hospital, College of Medicine, Korea University, 148 Gurodong-ro, Guro-Gu, Seoul, 152-703 South Korea

**Keywords:** Adverse pregnancy outcome, blood pressure, hypertension, preeclampsia

## Abstract

**Background:**

Hypertension has been known to increase the risk of obstetric complications. Recently, the American College of Cardiology endorsed lower thresholds for hypertension as systolic blood pressure of 130-139 mmHg or diastolic blood pressure 80-89 mmHg. However, there is a paucity of information regarding the impact of pre-pregnancy blood pressure on pregnancy outcomes. We aimed to evaluate the effect of pre-pregnancy blood pressure on maternal and neonatal complications.

**Methods:**

In this nationwide, population based study, pregnant women without history of hypertension and pre-pregnancy blood pressure < 140/90 mmHg were enrolled. The primary outcome of composite morbidity was defined as any of the followings: preeclampsia, placental abruption, stillbirth, preterm birth, or low birth weight.

**Results:**

A total of 375,305 pregnant women were included. After adjusting for covariates, the risk of composite morbidity was greater in those with stage I hypertension in comparison with the normotensive group (systolic blood pressure, odds ratio = 1.68, 95% CI: 1.59 – 1.78; diastolic blood pressure, odds ratio = 1.56, 95% CI: 1.42 – 1.72). There was a linear association between pre-pregnancy blood pressure and the primary outcome, with risk maximizing at newly defined stage I hypertension and with risk decreasing at lower blood pressure ranges.

**Conclusions:**

‘The lower, the better’ phenomenon was still valid for both maternal and neonatal outcomes. Our results suggest that the recent changes in diagnostic thresholds for hypertension may also apply to pregnant women. Therefore, women with stage I hypertension prior to pregnancy should be carefully observed for adverse outcomes.

## Background

Hypertension (HTN) is defined as an elevated blood pressure (BP) state, which can cause adverse effects if left untreated. Traditionally, guidelines have defined HTN as BP ≥ 140/90 mmHg, based on evidence from randomized clinical trials that treatment-induced BP reductions were beneficial at this threshold [1]. However, epidemiologic data have shown that the risk of cardiovascular disease already begins to rise above 120/80 mmHg [2]. Moreover, the SPRINT trial showed that even patients who were clinically believed to have prehypertension had increased risks of cardiovascular events [3]. These findings led to the lowering of BP thresholds for HTN diagnosis and treatment [2].

In the normal BP range, data regarding the impact of BP on cardiovascular risk have been inconsistent. Some studies have reported a linear relationship between BP and cardiovascular risk, showing progressive reductions in cardiovascular risk with decreasing BP [4, 5]. Others have proposed that a J-curve association exists between BP and cardiovascular risk specifically in those with coronary artery disease, suggesting that not only high, but low BP can also increase the risk of cardiovascular disease [6, 7].

In the case of pregnant women, HTN before pregnancy is a well-known risk factor for increased obstetric complications [8–10]. And blood pressure patterns in early pregnancy have been studied to be associated with an increased risk of hypertensive disorders during pregnancy which is common cause of maternal death [11, 12]. Having a persistently elevated diastolic BP (DBP ≥ 110 mmHg) despite therapy is a severe risk factor for pregnant women that affects pregnancy outcomes [13]. And whether lower thresholds for hypertension endorsed by the American College of Cardiology (ACC) can be applied to women has not yet been determined. Few studies have studied the impact of lower thresholds during pregnant period on diagnosis of preeclampsia and other pregnancy outcomes [14–16].

However, there is a paucity of information regarding the impact of pre-pregnancy BP on pregnancy outcomes. Moreover, the potential risk of stage I HTN before pregnancy has not been well examined. The current study was designed (1) to evaluate the effect of pre-pregnancy blood pressure on maternal and neonatal complications and (2) to determine whether there is a linear association between pre-pregnancy blood pressure and obstetric outcomes using a nationwide, population-based cohort.

## Methods

### Study population

Data on the study population was acquired from the Korean Health Insurance Review and Assessment (HIRA) service database. The Korean healthcare system is a single-payer system. Most of the population (97%) are registered with the National Health Insurance Service (NHIS), and all claims data are collected at HIRA. The database contains demographic, socioeconomic, diagnostic, procedural, and prescription information for its 50 million beneficiaries. Additionally, data from the bi-annual National Health Screening Examination (NHSE) provided by the NHIS, and the National Health Screening Program for Infants and Children (NHSP-IC) were used to assess pre-pregnancy BP and neonatal outcomes. The NHSP-IC, initiated in 2007, includes data on physical examination, anthropometric values, and results from developmental screening. All databases are open to researchers upon approval of their study protocols. The study was also approved by the Institutional Review Board of the Korean University Guro Hospital (No. 2020GR0105).

### Study design

The study population consisted of pregnant Korean women who met the following criteria: (1) singleton pregnancy; (2) delivery between 2007 and 2015; (3) participation in the NHSE within 6 months prior to pregnancy; and (4) pre-pregnancy BP < 140/90 mmHg. Women with multifetal pregnancies, pre-pregnant HTN, and those who lacked detailed clinical information were excluded from the study. Pre-pregnancy HTN was defined as having systolic BP (SBP) ≥ 140 mmHg, DBP ≥ 90 mmHg, or an International Classification of Diseases-10th Revision (ICD-10) code for HTN (I10 – I15). Stage I HTN was defined as having an SBP 130 – 139 mmHg or DBP 80 – 89 mmHg, according to the ACC guidelines [2]. Women were excluded from analysis for neonatal outcome if their offspring had not undergone at least one of the seven consecutive NHSP-IC health examinations.

### National health screening examination before pregnancy

Pre-pregnancy factors were collected using the NHSE database. The NHSE database is comprised of two components (health interview and health examination). The health interview questions contain information on demographics, socioeconomic status, and lifestyle. The smoking status before pregnancy was self-reported. The health examination includes physical examination and laboratory tests. Blood pressure was measured in the seated position using semi-automated sphygmomanometers, after at least 5 minutes of rest. Mean arterial pressure was calculated as 1/3(SBP) + 2/3(DBP). Blood samples were collected after at least 8 hours fasting, and aspartate aminotransferase (AST), alanine aminotransferase (ALT), and cholesterol levels were used for the analysis. The cohort profile and accuracy of the NHSE database have been described previously [17].

### Pregnancy and neonatal outcomes

Using the HIRA database, women with preeclampsia, placenta abruptio, or stillbirth during their pregnancy were identified by ICD-10 diagnostic codes. Data on neonatal outcomes, such as preterm birth and birth weight, were acquired from the NHSP-IC database. The primary outcome, which was composite morbidity, included preeclampsia, placenta abruptio, stillbirth, preterm birth, and low birth weight, [8, 18, 19] which have been reported to be increased in hypertensive pregnant women. Additional maternal outcomes, such as cesarean section, gestational diabetes mellitus (GDM), postpartum hemorrhage, and placenta previa, were also assessed. Finally, neonatal sex and birth weight were also included as neonatal outcomes.

### Statistical analysis

Shapiro-Wilk test was performed to assess normality distribution. The continuous variables are described as mean and standard deviation (SD), and compared by Student’s *t*-test or ANOVA for multiple group comparisons. The categorical variables are given as numbers and percentages and compared using the chi-square test. The study subjects were categorized into six groups by SBP, DBP, and me, and the risk of the outcomes was estimated for each group using an SBP of 110 – 119 mmHg, a DBP of 75 –59 mmHg, and a mean arterial pressure of 90 – 94 mmHg as references. Multivariate logistic regression analysis was used to estimate the adjusted odds ratios (OR) and 95% confidence intervals (CI). We included age, parity, obesity, high liver function test, high cholesterol, smoking, overt DM which are known predictors of preeclampsia and obstetrical complications as covariates [20–23] and that are differed when univariate analysis according to BP. Previous studies have demonstrated that the risk of developing HTN was higher in obese people, and that adiposity was related to the development of HTN. To evaluate this hypothesis, subgroup analysis was performed for different BMI values to adjust for the effect of obesity. The subjects were divided into three groups according to the Asian-Pacific cutoff points for BMI from the World Health Organization (WHO) [24]. For exploratory purposes, the relationships between BP and obstetric outcomes were also assessed by a restricted cubic splines [25]. The analyses were performed using SPSS version 23.0 (IBM Inc., Armonk, NY, USA), and a *P*-value of < 0.05 was considered statistically significant.

## Results

### Baseline characteristics

**Fig. 1:**
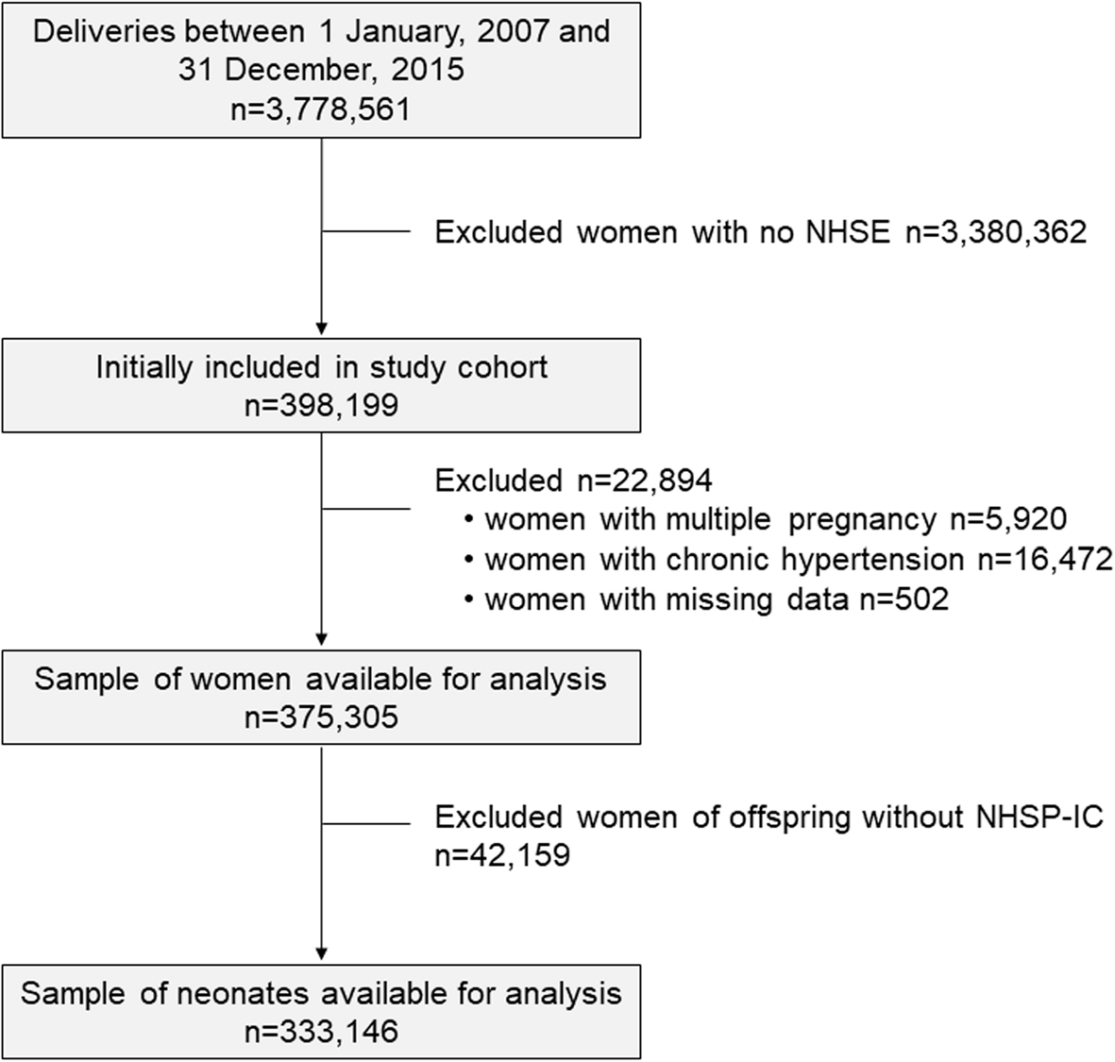
Flow chart of the participants

A total of 398,199 pregnant women who delivered between 2007 and 2015, and those who also underwent health examinations within 6 months prior to pregnancy were screened. Among the subjects screened, subjects with multi-fetal pregnancies (*n* = 5920), pre-existing HTN (*n* = 16,472), and those with missing data (*n* = 502) were excluded. The final cohort comprised of 375,305 subjects, as shown in Fig. 1. Women were excluded from the neonatal outcome analysis if their offspring had not undergone at least one of the seven consecutive NHSP-IC health examinations.

Table 1 describes the baseline characteristics of the study population. The mean pre-pregnancy SBP was 109.7 ± 10.4 mmHg and the DBP was 68.9 ± 7.7 mmHg. As expected, the study population was healthy young subjects with a low prevalence of comorbidities (diabetes, 4.03%; dyslipidemia, 2.52%; and current smoker, 2.87%). The baseline characteristics were compared between women with stage I HTN (SBP 130 – 139 mmHg or DBP 80 – 89 mmHg) and those with not (SBP <  130 mmHg and DBP <  80 mmHg). Women with stage I HTN had a higher body mass index (BMI), a higher frequency of comorbidities, and increased levels of AST, ALT, and total cholesterol compared to normotensive women (Tables 2, 3).

**Table 1:** Baseline characteristics of the study population

Characteristics	Values (***n*** = 375,305)
Age, years (n)	30.7 ± 3.7
Primiparity	253,762 (67.6)
**Pre-pregnancy measurements (n)**	
Body mass index (kg/m^2^)	21.0 ± 2.8
Obesity (BMI ≥ 25)	31,445 (8.4)
Systolic blood pressure (mmHg)	109.7 ± 10.4
Diastolic blood pressure (mmHg)	68.9 ± 7.7
**Pre-pregnancy comorbidities**	
Diabetes	15,129 (4.0)
Dyslipidemia (cholesterol ≥240)	9472 (2.5)
Current smoker	10,766 (2.9)
**Pre-pregnancy laboratory findings**	
AST	19.1 ± 9.6
ALT	15.3 ± 14.7
High LFT (AST or ALT ≥80)	1798 (0.5)
Cholesterol	175.5 ± 30.0

Data are presented as number (%) or mean ± SE

Dyslipidemia: total cholesterol ≥240 mg/dL

Abbreviations: *ALT* Alanine aminotransferase, *AST* Aspartate aminotransferase

**Table 2:** Baseline characteristics of the study population by pre-pregnancy systolic blood pressure

Characteristics	< 130 (***n*** = 357,761)	130 – 139 (***n*** = 17,544)	*P*-value
**Age, years (n)**	30.7 ± 3.6	31.0 ± 4.0	<.0001
**Primiparity**	241,842 (67.6)	11,920 (67.9)	0.3408
**Pre-pregnancy anthropometric measurements (n)**			
Body mass index (kg/m^2^)	20.9 ± 2.7	22.7 ± 3.9	<.0001
Obesity (BMI ≥ 25)	27,300 (7.6)	4145 (23.6)	<.0001
Systolic blood pressure (mmHg)	108.6 ± 9.3	131.9 ± 2.7	<.0001
Diastolic blood pressure (mmHg)	68.4 ± 7.4	79.3 ± 5.5	<.0001
**Pre-pregnancy comorbidities**			
Diabetes	14,368 (4.0)	761 (4.3)	<.05
Dyslipidemia (Cholesterol ≥240)	8680 (2.4)	792 (4.5)	<.0001
Current smoker before pregnancy	10,021 (2.8)	745 (4.3)	<.0001
**Pre-pregnancy laboratory findings**			
AST	19.1 ± 9.6	20.2 ± 9.3	<.0001
ALT	15.1 ± 14.7	17.7 ± 15.6	<.0001
High LFT (AST or ALT ≥80)	1626 (0.5)	172 (1.0)	<.0001
Cholesterol	175.2 ± 29.6	182.2 ± 36.7	<.0001

Data are presented as number (%) or mean ± SE

Dyslipidemia: total cholesterol ≥240 mg/dL

Abbreviations: *ALT* Alanine aminotransferase, *AST* Aspartate aminotransferase

**Table 3:** Baseline characteristics of the study population by pre-pregnancy diastolic blood pressure

Characteristics	< 80 (***n*** = 317,818)	80 – 89 (***n*** = 57,487)	P-value
**Age, years (n)**	30.7 ± 3.6	30.8 ± 3.8	0.1392
**Primiparity**	214,446 (67.5)	39,316 (68.4)	<.0001
**Pre-pregnancy anthropometric measurements (n)**			
Body mass index (kg/m^2^)	20.9 ± 2.6	21.8 ± 3.4	<.0001
Obesity (BMI ≥ 25)	22,770 (7.2)	8675 (15.1)	<.0001
Systolic blood pressure (mmHg)	107.7 ± 9.4	121.2 ± 7.6	<.0001
Diastolic blood pressure (mmHg)	66.7 ± 6.1	81.1 ± 2.3	<.0001
**Pre-pregnancy comorbidities**			
Diabetes	12,638 (4.0)	2491 (4.3)	<.0001
Dyslipidemia (Cholesterol ≥240)	7448 (2.3)	2024 (3.5)	<.0001
Current smoker before pregnancy	8737 (2.8)	2029 (3.5)	<.0001
**Pre-pregnancy laboratory findings**			
AST	19.0 ± 9.6	19.7 ± 9.1	<.0001
ALT	15.1 ± 14.7	16.4 ± 14.7	<.0001
High LFT (AST or ALT ≥80)	1352 (0.4)	446 (0.8)	<.0001
Cholesterol	174.8 ± 29.8	179.2 ± 31.0	<.0001

Data are presented as number (%) or mean ± SE.

Dyslipidemia: total cholesterol ≥240 mg/dL.

Abbreviations: *ALT* Alanine aminotransferase, *AST* Aspartate aminotransferase

### Pregnancy outcomes

**Fig. 2:**
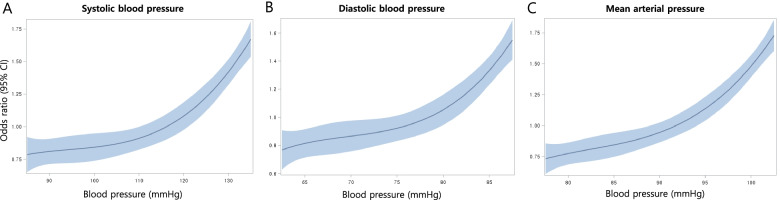
The adjusted odds ratio of composite morbidity according to pre-pregnancy blood pressure

Pregnancy and neonatal outcomes were assessed according to the pre-pregnancy BP categories. Having a pre-pregnancy SBP ≥ 130 mmHg significantly increased the risk for composite morbidity compared to an SBP of 110 – 119 mmHg (OR = 1.681, 95% CI: 1.586 – 1.783). Having a pre-pregnancy DBP (≥ 85 mmHg) also increased the risk of the primary outcome compared to a DBP of 75 – 79 mmHg (OR = 1.560, 95% CI: 1.420 – 1.715). Even at normal and elevated BP (< 130/80 mmHg), lower BP was associated with a significantly lower risk of the primary outcome. This linear relationship between BP and composite morbidity was consistent in the SBP, DBP, and mean arterial pressure categories, and also after adjusting for covariates (Tables 4, 5 and 6). The relationship between the risk of the composite morbidity and BP values is visualized in Fig. 2 using restricted cubic splines.

**Table 4:** Adjusted odds ratio of obstetric outcomes across systolic blood pressure subgroups

	< 90 (***n*** = 3414)	90 – 99 (***n*** = 40,468)	100 – 109 (***n*** = 109,985)	110 – 119 (***n*** = 139,082)	120 – 129 (***n*** = 64,812)	130 – 139 (***n*** = 17,544)
Preeclampsia	0.383 (0.249,0.590)	0.456 (0.404,0.515)	0.625 (0.582,0.672)	reference	1.504 (1.411,1.602)	2.734 (2.523,2.961)
Placenta abruptio	0.911 (0.513,1.617)	1.025 (0.857,1.225)	1.086 (0.957,1.232)	reference	1.107 (0.956,1.282)	1.039 (0.809,1.334)
Stillbirth	2.038 (0.491,8.464)	1.606 (0.915,2.819)	0.732 (0.433,1.239)	reference	0.973 (0.554,1.707)	1.671 (0.800,3.492)
	**(*n*** **= 2983)**	**(*n =*** **35,623)**	**(*n*** **= 97,608)**	**(*n*** **= 123,852)**	**(*n =*** **57,911)**	**(*n*** **= 15,709)**
Preterm birth	0.912 (0.708,1.173)	0.865 (0.796,0.940)	0.973 (0.920,1.030)	reference	1.116 (1.048,1.189)	1.322 (1.200,1.457)
LBW	0.902 (0.730,1.115)	0.865 (0.807,0.927)	0.936 (0.892,0.982)	reference	1.135 (1.076,1.197)	1.307 (1.201,1.421)
Composite morbiditya	0.795 (0.668,0.946)	0.802 (0.759,0.847)	0.884 (0.852,0.918)	reference	1.199 (1.152,1.248)	1.681 (1.586,1.783)

Data are presented as odds ratio and 95% confidential intervals of each obstetric outcomes across blood pressure subgroups

Adjusted for.

Abbreviations: *LBW* Low birth weight

^a^Includes any of the following: preeclampsia, placenta abruptio, stillbirth, preterm birth, low birth weight

**Table 5:** Adjusted odds ratio of obstetric outcomes across diastolic blood pressure subgroups

	< 65 (***n*** = 115,678)	65 – 69 (***n*** = 45,474)	70 – 74 (***n =*** 123,087)	75 – 79 (***n*** = 33,579)	80 – 84 (***n =*** 50,349)	85 – 89 (***n*** = 7138)
Preeclampsia	0.463 (0.421,0.510)	0.635 (0.569,0.708)	0.799 (0.734,0.871)	reference	1.395 (1.276,1.526)	2.423 (2.140,2.743)
Placenta abruptio	1.045 (0.859,1.272)	1.051 (0.839,1.316)	0.999 (0.822,1.214)	reference	1.170 (0.943,1.453)	1.191 (0.815,1.740)
Stillbirth	0.831 (0.427,1.618)	0.876 (0.404,1.896)	0.559 (0.278,1.124)	reference	1.307 (0.650,2.627)	1.053 (0.296,3.745)
	**(*n*** **= 102,370)**	**(*n*** **= 39,664)**	**(*n*** **= 110,087)**	**(*n =*** **29,539)**	**(*n =*** **45,231)**	**(*n*** **= 6255)**
Preterm birth	0.900 (0.826,0.979)	0.940 (0.851,1.037)	0.960 (0.884,1.044)	reference	1.077 (0.982,1.182)	1.345 (1.152,1.569)
LBW	0.819 (0.763,0.879)	0.847 (0.780,0.920)	0.886 (0.826,0.949)	reference	1.012 (0.936,1.094)	1.216 (1.064,1.390)
Composite morbidity^a^	0.776 (0.735,0.820)	0.824 (0.773,0.879)	0.899 (0.852,0.948)	reference	1.141 (1.076,1.209)	1.560 (1.420,1.715)

Data are presented as odds ratio and 95% confidential intervals of each obstetric outcomes across blood pressure subgroups.

Adjusted for age, parity, obesity, high LFT, high cholesterol, smoker, overt DM

Abbreviations: *LBW* Low birth weight

^a^Includes any of the following: preeclampsia, placenta abruptio, stillbirth, preterm birth, low birth weight

**Table 6:** Adjusted odds ratio of obstetric outcomes across mean arterial pressure subgroups

	< 80 (***n*** = 129,158)	80 – 84 (***n*** = 114,661)	85 – 89 (***n*** = 53,357)	90 – 94 (***n*** = 55,844)	95 – 99 (***n*** = 17,783)	≥ 100 (***n*** = 4804)
Preeclampsia	0.384 (0.355,0.415)	0.618 (0.575,0.664)	0.739 (0.681,0.803)	reference	1.690 (1.547,1.846)	2.365 (2.085,2.683)
Placenta abruptio	0.949 (0.812,1.109)	0.920 (0.785,1.080)	0.962 (0.799,1.159)	reference	0.969 (0.746,1.260)	1.284 (0.860,1.917)
Stillbirth	0.904 (0.519,1.575)	0.650 (0.355,1.188)	0.838 (0.425,1.649)	reference	1.230 (0.537,2.818)	1.578 (0.464,5.368)
	**(*n*** **= 113,943)**	**(*n =*** **102,146)**	**(*n*** **= 47,110)**	**(*n*** **= 49,886)**	**(*n =*** **15,820)**	**(*n*** **= 4241)**
Preterm birth	0.872 (0.814,0.935)	0.932 (0.869,0.999)	0.957 (0.882,1.038)	reference	1.202 (1.082,1.337)	1.418 (1.198,1.677)
LBW	0.804 (0.759,0.852)	0.860 (0.811,0.912)	0.909 (0.850,0.974)	reference	1.091 (0.996,1.196)	1.277 (1.100,1.482)
Composite morbidity^a^	0.726 (0.695,0.759)	0.825 (0.790,0.863)	0.884 (0.839,0.931)	reference	1.313 (1.231,1.401)	1.720 (1.556,1.901)

Data are presented as odds ratio and 95% confidential intervals of each obstetric outcomes across blood pressure subgroups.

Adjusted for age, parity, obesity, high LFT, high cholesterol, smoker, overt DM.

Abbreviations: *LBW* Low birth weight

^a^Includes any of the following: preeclampsia, placenta abruptio, stillbirth, preterm birth, low birth weight

### Subgroup analysis

**Fig. 3:**
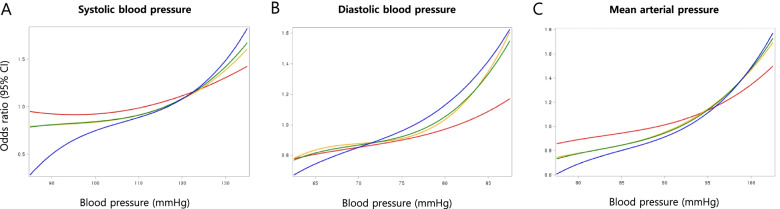
Blood pressure and risk of composite morbidity. Red: underweight (BMI < 18.5 kg/m^2^). Orange: normal (BMI < 18.5-22.9 kg/m^2^). Blue: overweight/obese (BMI ≥ 23 kg/m^2^). Green: total study population

Subgroup analysis was performed using pre-pregnancy BMIs. The subjects were categorized into three groups: underweight (< 18.5 kg/m^2^), normal (18.5 – 22.9 kg/m^2^), and overweight/obese (≥ 23 kg/m^2^). The linear relationship between pre-pregnancy BP and composite morbidity was consistent across all BMI subgroups and BP components, but the rate of increase was greater in overweight or obese women (pre-pregnancy BMI ≥ 23 kg/m^2^) (Fig. 3).

### Pregnancy and neonatal outcomes according to pre-pregnancy BP

**Fig. 4:**
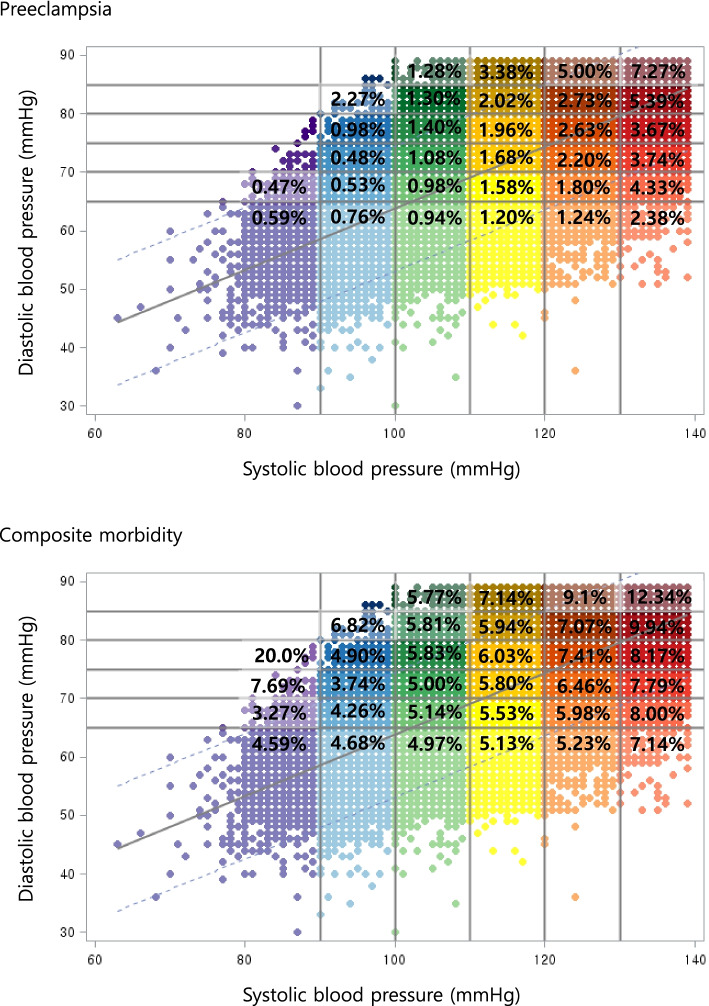
**1**. Distribution of systolic and diastolic blood pressure before pregnancy and rates of preeclampsia. **2**. Distribution of systolic and diastolic blood pressure before pregnancy and rates of composite morbidity

The incidence rates of the primary outcome and its components, and additional maternal outcomes according to BP values are shown in Tables 7, 8 and 9. Higher SBP was associated with increased rates of cesarean sections, preeclampsia, and GDM, whereas no significant differences were observed for postpartum hemorrhage, placental abruption, or placenta previa. For neonatal outcomes, significant increases in preterm birth, and low birth weight were observed in patients with higher SBP. The association between BP and outcomes was also consistent for the DBP and mean arterial pressure, except for postpartum hemorrhage, showing significantly increased events at higher DBP and mean arterial pressure. Figure 4 shows the incidence of the primary outcome and preeclampsia according to baseline systolic and diastolic BPs. Both systolic and diastolic BP was associated with the incidence of preeclampsia, with the highest incidence observed at SBPs of 130 – 139 mmHg and DBPs of 85 – 89 mmHg.

**Table 7:** Pregnancy and neonatal outcomes of the study population by pre-pregnancy systolic blood pressure

	< 90 (***n =*** 3414)	90 – 99 (***n =*** 40,468)	100 – 109 (***n =*** 109,985)	110 – 119 (***n =*** 139,082)	120 – 129 (***n =*** 64,812)	130 – 139 (***n =*** 17,544)	*P*-value^a^
Pregnancy outcome							
Cesarean section (n, %)	1084 (31.8)	12,655 (31.3)	35,614 (32.4)	46,768 (33.6)	23,323 (36.0)	6827 (38.9)	< .0001
Preeclampsia (n, %)	21 (0.6)	297 (0.7)	1121 (1.0)	2344 (1.7)	1710 (2.6)	910 (5.2)	< .0001
GDM (n, %)	96 (2.8)	1094 (2.7)	2815 (2.6)	3975 (2.9)	2052 (3.2)	732 (4.2)	< .0001
PPH (n, %)	310 (9.1)	3377 (8.3)	9140 (8.3)	11,553 (8.3)	5366 (8.3)	1423 (8.1)	0.5924
Placenta abruptio (n,%)	12 (0.4)	158 (0.4)	453 (0.4)	528 (0.4)	274 (0.4)	71 (0.4)	0.7036
Placenta previa (n, %)	39 (1.1)	428 (1.1)	1185 (1.1)	1498 (1.1)	696 (1.1)	199 (1.1)	0.9755
Stillbirth (n, %)	2 (0.1)	18 (0.0)	22 (0.0)	38 (0.0)	18 (0.0)	9 (0.1)	0.056
Neonatal outcomes							
	**(*n =*** **2983)**	**(*n =*** **35,623)**	**(*n =*** **97,608)**	**(*n =*** **123,852)**	**(*n =*** **57,911)**	**(*n =*** **15,709)**	
Neonatal sex–male (n, %)	1550 (52.0)	18,387 (51.6)	49,915 (51.4)	63,679 (51.4)	29,577 (51.1)	8083 (51.5)	0.6175
Preterm birth (n, %)	63 (2.1)	711 (2.0)	2175 (2.2)	2872 (2.3)	1523 (2.6)	504 (3.21	< .0001
Birth weight (g)	3194 (0.4)	3216 (0.4)	3217 (0.5)	3216 (0.5)	3211 (0.5)	3215 (0.5)	<.05
LBW (n, %)	90 (3.0)	1023 (2.9)	2996 (3.1)	4063 (3.3)	2154 (3.7)	672 (4.3)	< .0001
Composite morbidity^b^	136 (4.6)	1634 (4.6)	4900 (5.1)	7100 (5.7)	4007 (6.9)	1537 (9.8)	< .0001

Data are presented as number (%)

^a^compared using the chi-square test

Abbreviations: *GDM* Gestational diabetes mellitus, *LBW* Low birth weight, *PPH* Postpartum hemorrhage

^b^Includes any of the following: preeclampsia, placenta abruptio, stillbirth, preterm birth, low birth weight

**Table 8:** Pregnancy and neonatal outcomes of the study population by pre-pregnancy diastolic blood pressure

	< 65 (***n =*** 115,678)	65 – 69 (***n =*** 45,474)	70 – 74 (***n =*** 123,087)	75 – 79 (***n =*** 33,579)	80 – 84 (***n =*** 50,349)	85 – 89 (***n =*** 7138)	*P*-value^a^
Pregnancy outcome							
Cesarean section (n, %)	36,961 (32.0)	15,061 (33.1)	41,489 (33.7)	11,742 (35.0)	18,145 (36.0)	2873 (40.3)	< .0001
Preeclampsia (n, %)	1067 (0.9)	597 (1.3)	2041 (1.7)	738 (2.2)	1543 (3.1)	417 (5.8)	< .0001
GDM (n, %)	2940 (2.5)	1429 (3.1)	3290 (2.7)	1206 (3.6)	1588 (3.2)	311 (4.4)	< .0001
PPH (n, %)	9455 (8.2)	3911 (8.6)	10,141 (8.2)	2954 (8.8)	4037 (8.0)	671 (9.4)	< .0001
Placenta abruptio (n,%)	457 (0.4)	184 (0.4)	466 (0.4)	130 (0.4)	225 (0.5)	34 (0.5)	0.361
Placenta previa (n, %)	1247 (1.1)	487 (1.1)	1326 (1.1)	388 (1.2)	510 (1.0)	87 (1.2)	0.3873
Stillbirth (n, %)	32 (0.0)	14 (0.0)	23 (0.0)	12 (0.0)	23 (0.1)	3 (0.0)	0.0608
Neonatal outcomes							
	**(*n =*** **102,370)**	**(*n =*** **39,664)**	**(*n =*** **110,087)**	**(*n =*** **29,539)**	**(*n =*** **45,231)**	**(*n =*** **6255)**	
Neonatal sex–male (n, %)	52,720 (51.5)	20,454 (51.6)	56,627 (51.4)	15,045 (51.0)	23,081 (51.0)	3264 (52.2)	0.1861
Preterm birth (n, %)	2215 (2.2)	911 (2.3)	2566 (2.3)	733 (2.5)	1206 (2.7)	217 (3.5)	< .0001
Birth weight (g)	3215 (0.4)	3221 (0.5)	3215 (0.4)	3211 (0.5)	3212 (0.5)	3215 (0.5)	0.0662
LBW (n, %)	3096 (3.0)	1251 (3.2)	3589 (3.3)	1094 (3.7)	1684 (3.7)	284 (4.5)	< .0001
Composite morbidity^b^	5028 (4.9)	2100 (5.3)	6308 (5.7)	1918 (6.5)	3318 (7.3)	642 (10.3)	< .0001

Data are presented as number (%)

^a^compared using the chi-square test

Abbreviations: *GDM* Gestational diabetes mellitus, *LBW* Low birth weight, *PPH* Postpartum hemorrhage

^b^Includes any of the following: preeclampsia, placental abruption, stillbirth, preterm birth, low birth weight

**Table 9:** Pregnancy and neonatal outcomes of the study population by pre-pregnancy mean arterial pressure

	< 80 (***n =*** 129,158)	80 – 84 (***n =*** 114,661)	85 – 89 (***n =*** 53,357)	90 – 94(***n =*** 55,844)	95 – 99 (***n =*** 17,783)	≥ 100 (***n =*** 4804)	*P*-value^a^
Pregnancy outcome							
Cesarean section (n, %)	41,227 (31.9)	38,060 (33.3)	18,210 (34.1)	20,015 (35.8)	6769 (38.1)	1990 (41.4)	< .0001
Preeclampsia (n, %)	1162 (0.9)	1702 (1.5)	982 (1.8)	1416 (2.5)	816 (4.6)	325 (6.8)	< .0001
GDM (n, %)	3343 (2.6)	3070 (2.7)	1645 (3.1)	1758 (3.2)	719 (4.0)	229 (4.8)	< .0001
PPH (n, %)	10,659 (8.3)	9506 (8.3)	4492 (8.4)	4587 (8.2)	1466 (8.2)	459 (9.6)	< .05
Placenta abruptio (n,%)	509 (0.4)	437 (0.4)	215 (0.4)	234 (0.4)	74 (0.4)	27 (0.6)	0.437
Placenta previa (n, %)	1373 (1.1)	1251 (1.1)	555 (1.0)	617 (1.1)	192 (1.1)	57 (1.2)	0.8256
Stillbirth (n, %)	38 (0.0)	24 (0.0)	15 (0.0)	19 (0.0)	8 (0.0)	3 (0.1)	0.2569
Neonatal outcomes							
	**(*n =*** **113,943)**	**(*n =*** **102,146)**	**(*n =*** **47,110)**	**(*n =*** **49,886)**	**(*n =*** **15,820)**	**(*n =*** **4241)**	
Neonatal sex–male (n, %)	58,650 (51.5)	52,605 (51.5)	24,226 (51.4)	25,356 (50.8)	8129 (51.4)	2225 (52.5)	0.1053
Preterm birth (n, %)	2450 (2.2)	2354 (2.3)	1131 (2.4)	1260 (2.5)	493 (3.1)	160 (3.8)	< .0001
Birth weight (g)	3216 (0.4)	3217 (0.4)	3213 (0.5)	3212 (0.5)	3212 (0.5)	3212 (0.5)	0.1802
LBW (n, %)	3426 (3.0)	3269 (3.2)	1599 (3.4)	1855 (3.7)	646 (4.1)	203 (4.8)	< .0001
Composite morbidity^b^	5535 (4.9)	5652 (5.5)	2820 (6.0)	3381 (6.8)	1425 (9.0)	501 (11.8)	< .0001

Data are presented as number (%).

^a^compared using the chi-square test

Abbreviations: *GDM* Gestational diabetes mellitus, *LBW* Low birth weight, *PPH* Postpartum hemorrhage

^b^Includes any of the following: preeclampsia, placenta abruptio, stillbirth, preterm birth, low birth weight

## Discussion

### Principle Findings

The current analysis of a large number of pregnant women found that (1) women with pre-pregnancy stage I HTN (SBP ≥ 130 mmHg or DBP ≥ 85 mmHg) had a significantly increased risk of composite morbidity; (2) there was a linear relationship between BP and risk of composite morbidity, with a progressive decrease in risk at lower BP observed even in women with normal BP; (3) the relationship between pre-pregnancy BP and composite morbidity was consistent across various BMI subgroups, with the steepest slope observed in overweight/obese women; and (4) both SBP and DBP were associated with increased risks for preeclampsia and composite morbidity.

### Results

The risk of cardiovascular disease is known to increase with increases in BP, and lowering BP reduces this risk in hypertensive patients [26, 27]. Traditionally, the threshold at which BP interventions have been beneficial has been 140/90 mmHg. Those with prehypertension or elevated BP, are at an increased risk of developing HTN, but no benefit has been observed with treatment. However, recent ACC/AHA guidelines changed the HTN threshold to **≥**130/80 mmHg as a result of the SPRINT trial [2]. It is still debatable whether subjects with borderline BP will benefit from intensive treatment, and whether this threshold can also be applied to younger, healthier subjects. Using a large, nationwide database, we were able to analyze the effect of stage I hypertension on maternal and neonatal outcomes in young, healthy women.

The obstetric risk of stage I HTN was also reported in a study by Reddy et al. In this study, stage I HTN during pregnancy was associated with an increased risk of preeclampsia, preterm birth and adverse perinatal outcomes, showing the clinical risk of stage I HTN during pregnancy [16]. And other studies showed that use of newly endorsed lower BP thresholds has been shown to better identify the risk of preeclampsia [14, 15], gestational diabetes and preterm birth [28]. In the current study, we evaluated the significance of pre-pregnancy stage I HTN and demonstrated that having elevated BP prior to pregnancy significantly affected maternal and neonatal outcomes and that the risk of preeclampsia also increased.

Until now, the clinical significance of stage I HTN in the pre-pregnancy period has not been well studied. In the current study, pre-pregnancy stage I HTN significantly increased the composite morbidity of the mother and fetus, increasing the risk of outcomes, such as preeclampsia, placental abruption, stillbirth, preterm birth, and low birth weight. The incidence of composite morbidity and preeclampsia was high, at 12 and 7%, respectively, in women with stage I HTN. As the current guidelines have yet to identify this group as having increased risk, we believe that these women should be intensively monitored during pregnancy and that they may be candidates for prevention treatment, such as aspirin prophylaxis.

As pregnancy is the window period used to assess future ardiovascular risk [29], we tried to evaluate the occurrence of pregnancy complications in women with BP < 140/90 mmHg. In pregnant women, the lowest obstetric risk was observed in the lowest BP category, showing a ‘the lower, the better’ association.

### Clinical and Research implications

In this study, we defined composite morbidity as outcomes including preeclampsia, placental abruption, stillbirth, preterm birth, and low birth weight. These maternal and neonatal outcomes are well-known adverse events associated with chronic HTN [8, 18, 19]. Theoretically, elevated BP during pregnancy is a risk factor for adverse pregnancy outcomes because several conditions, such as preterm birth, small-for-gestational age, uteroplacental insufficiency, and gestational HTN or preeclampsia, are related to elevated BP. Our results suggest that the recent changes in diagnostic thresholds for HTN may also apply to pregnant women. Although the newly endorsed stage I HTN before pregnancy showed a worsening of pregnancy outcome, in the current study we did not confirm that the use of antihypertensive drugs in stage I HTN improved the prognosis. On this basis, there is a need for future prospective clinical trials to evaluate the benefits of intensive preventive treatment.

Another issue to consider is whether different BP components, systolic, diastolic or mean arterial pressure, correlate better with adverse outcomes. In non-pregnant adults, systolic BP is the main component in diagnosing and treating HTN. However, in pregnant women, DBP has also been considered to be important in diagnosing and initiating treatment [30]. In our analysis, all systolic, diastolic, and mean arterial pressures were significantly associated with composite morbidity.

In a subgroup analysis according to pre-pregnancy BMI, the linear relationship between BP and composite morbidity was consistent across the various BMI groups. Previous studies have demonstrated that the risk of developing HTN was higher in obese people, and BMI itself can affect pregnancy outcomes [31–34]. In the overweight/obese group, defined as a pre-pregnancy BMI of ≥23 kg/m^2^, a steep slope was observed between pre-pregnancy BP and composite morbidity. This finding suggests the increased effects of BP changes on outcomes in obese, pregnant women. Therefore, overweight women with stage I HTN prior to pregnancy should be carefully observed for adverse outcomes.

### Strengths and Limitations

Our study had several strengths and limitations. This is a large-scale study that systematically evaluated the obstetrical outcome after pregnancy according to the pre-pregnancy BP category. Women of childbearing age are usually healthy and young, and it is rare to check their BP or undergo health check-ups. Therefore, it is difficult to conduct a study to confirm the outcome of BP before pregnancy. However, due to a government-paid, bi-annual health screening examination, we were able to acquire data on pre-pregnancy BP and assess its correlation with composite morbidity in a large cohort. In addition, systolic, diastolic, and mean arterial pressure were separately analyzed to examine the effect each BP component had on the outcome. The study also has a limitation in its retrospective design. As it is not mandatory to undergo health examinations, we cannot exclude the possibility of selection bias. Furthermore, incorrect or failure to input appropriate diagnostic codes might have led to an underestimation of events. And we could not handle all the predisposing conditions to preeclampsia and adverse pregnancy outcomes, medication history and social economic status that may affect results. A well-designed prospective study is needed to better assess the relationship between pre-pregnancy BP and outcomes. Pre-pregnancy stage I HTN was associated with an increased risk of maternal and neonatal adverse outcomes. ‘The lower, the better’ phenomenon was still valid for both maternal and neonatal outcomes.

## Conclusions

There was a linear association between pre-pregnancy blood pressure and the maternal and neonatal outcomes, with risk maximizing at newly defined stage I hypertension and with risk decreasing at lower blood pressure ranges. Our results suggest that the recent changes in diagnostic thresholds for HTN may also apply to pregnant women. Therefore, women with stage I HTN prior to pregnancy should be carefully observed for adverse outcomes.

## Data Availability

The data that support the findings of this study are available from the National Health Insurance Service (NHIS), but restrictions apply to the availability of these data, which were used under license for the current study and so are not publicly available. Data are however available from the author (Geum Joon Cho) upon reasonable request and with permission of the NHIS. The results do not necessarily represent the opinion of the National Health Insurance Corporation.

## Abbreviations

HTN: Hypertension
BP: Blood pressure
DBP: Diastolic blood pressure
HIRA: Health Insurance Review and Assessment
NHIS: National Health Insurance Service
NHSE: National Health Screening Examination
NHSP-IC: National Health Screening Program for Infants and Children
ICD-10: International Classification of Diseases-10th Revision
SBP: Systolic blood pressure
ACC: Amerian College of Cardiology
AST: Aspartate aminotransferase
ALT: Alanine aminotransferase
GDM: Gestational diabetes mellitus
SD: Standard deviation
OR: Odds ratio
CI: Confidence intervals
WHO: World Health Organization
BMI: Body mass index
